# Eicosapentaenoic acid prevents the progression of intracranial aneurysms in rats

**DOI:** 10.1186/s12974-020-01802-8

**Published:** 2020-04-24

**Authors:** Yu Abekura, Isao Ono, Akitsugu Kawashima, Katsumi Takizawa, Hirokazu Koseki, Haruka Miyata, Kampei Shimizu, Mieko Oka, Mika Kushamae, Susumu Miyamoto, Hiroharu Kataoka, Akira Ishii, Tomohiro Aoki

**Affiliations:** 1 Department of Molecular Pharmacology, Research Institute, National Cerebral and Cardiovascular Center, Suita, Osaka, 564-8565 Japan; 2 Department of Neurosurgery, Kyoto University Graduate School of Medicine, Kyoto, Japan; 3 Core Research for Evolutional Science and Technology (CREST) from Japan Agency for Medical Research and Development (AMED), National Cerebral and Cardiovascular Center, Osaka, Japan; 4 Department of Neurosurgery, Tokyo Women’s Medical University Yachiyo Medical Center, Chiba, Japan; 5 Department of Neurosurgery, Asahikawa Red Cross Hospital, Hokkaido, Japan; 6 Department of Neurosurgery, The JIKEI University Hospital, Tokyo, Japan; 7 Department of Neurosurgery, Shiga University of Medical Science, Shiga, Japan; 8 Department of Neurosurgery, Tokyo Women’s Medical University, Tokyo, Japan; 9 Department of Neurosurgery, Showa University, Tokyo, Japan

**Keywords:** Intracranial aneurysm, Eicosapentaenoic acid, GPR120

## Abstract

**Background:**

As subarachnoid hemorrhage due to rupture of an intracranial aneurysm (IA) has quite a poor outcome despite of an intensive medical care, development of a novel treatment targeting unruptured IAs based on the correct understanding of pathogenesis is mandatory for social health.

**Methods:**

Using previously obtained gene expression profile data from surgically resected unruptured human IA lesions, we selected G-protein coupled receptor 120 (GPR120) as a gene whose expression is significantly higher in lesions than that in control arterial walls. To corroborate a contribution of GPR120 signaling to the pathophysiology, we used an animal model of IAs and examine the effect of a GPR120 agonist on the progression of the disease. IA lesion was induced in rats through an increase of hemodynamic stress achieved by a one-sided carotid ligation and induced hypervolemia. Eicosapentaenoic acid (EPA) was used as an agonist for GPR120 in this study and its effect on the size of IAs, the thinning of media, and infiltration of macrophages in lesions were examined.

**Result:**

EPA administered significantly suppressed the size of IAs and the degenerative changes in the media in rats. EPA treatment also inhibited infiltration of macrophages, a hallmark of inflammatory responses in lesions. In in vitro experiments using RAW264.7 cells, pre-treatment of EPA partially suppressed lipopolysaccharide-induced activation of nuclear factor-kappa B and also the transcriptional induction of monocyte chemoattractant protein 1 (MCP-1), a major chemoattractant for macrophages to accumulate in lesions. As a selective agonist of GPR120, TUG-891, could reproduce the effect of EPA in RAW264.7 cells, EPA presumably acted on this receptor to suppress inflammatory responses. Consistently, EPA remarkably suppressed MCP-1 expression in lesions, suggesting the in vivo relevance of in vitro studies.

**Conclusions:**

These results combined together suggest the potential of the medical therapy targeting GPR120 or using EPA to prevent the progression of IAs.

## Background

Subarachnoid hemorrhage (SAH) mainly caused by the rupture of an intracranial aneurysm (IA) has the mortality rate as high as 50% within 1 month from the onset [1, 2], making this disease being one of the most serious health problems. The incidence of SAH is 9/100,000 persons per year worldwide [3] but, in Finland and Japan, the incidence reaches 20~30/100,000 persons per year [3, 4]. Because the prevalence of unruptured IAs in general population is high, 2–4% [5], and IA can cause the lethal SAH after rupture, the preemptive treatment of unruptured IAs to prevent the onset of SAH is mandatory for social health. Although IAs are often found before rupture during brain check or so, surgical interventions such as clipping and coil embolization are applied only to the selected patients whose IAs are considered at high risk of rupture depending on the size, shape, and location of IAs [6]. In addition, surgical interventions have the potential risk of complication and thus occasionally result in unfavorable outcome. Moreover, because of no effective medical treatment available, patients without surgical indication or followed up without surgical interventions do not have any choice but to rule out the progression of IAs by a long-term follow-up. The establishment of less-invasive medical treatment to prevent the progression and the rupture of IAs is therefore a matter to be urgently addressed.

Considered with the current situation surrounding IA treatment as described above, the inquisition of a novel therapeutic target to develop drugs to treat IAs, especially to prevent the progression, is crucial for social health. The experimental studies have clarified the crucial role of nuclear factor-kappa B (NF-κB)-mediated inflammatory responses in the pathogenesis of IAs [7–9]. The pharmacological measures interfering NF-κB pathway may thus become a reasonable strategy as the treatment of IAs. To explore a therapeutic target, we selected G-protein coupled receptors (GPCRs), that were upregulated in IA walls compared with those in the control arterial walls, as candidates [10] because GPCRs are on the top of signal transduction cascades and, thereby, inhibition of these receptors is presumably efficient and effective. We, in the present study, have then verified the potential of one of such upregulated GPCRs in lesions, GPR120, as a therapeutic target because GPR120, a receptor of ω3 fatty acids, could inhibit NF-κB activation [11]. Stimulation of GPR120 with its ligand induces the formation of the GPR120/β-arrestin-2 complex and then β-arrestin-2 traps transforming growth factor-β (TGF-β) activated kinase 1 binding protein (TAB1), leading to the inhibition of TGF-β activated kinase 1(TAK1) which activates inhibitor of κB kinase subunit β (IKKβ) [11, 12].

## Methods

### Human specimen and immunohistochemistry

Human IA samples and control arterial walls (superficial temporal artery or middle meningeal artery) were dissected during microsurgical clipping of unruptured IAs with the written informed consent. Dissected specimens were fixed in formalin solution and embedded in paraffin. Four-micrometer-thick slices were then prepared for histopathological and immunohistochemical analyses. After deparaffinization and blocking with 3% donkey serum (Jackson ImmunoResearch, West Grove, PA), slices were incubated with primary antibodies overnight at 4 °C in a humidified chamber, followed by incubation with secondary antibodies conjugated with fluorescence dye (Jackson ImmunoResearch) for additional 1 h at room temperature in a humidified chamber. Finally, fluorescent images were acquired on a confocal fluorescence microscope system (FV1000, Olympus, Tokyo, Japan). Scanning was performed using a UPLSAPO 40×/NA 0.9 objective (Olympus) at a resolution of 1024 × 1024 pixels with a field of view of 317 μm square. Maximal intensity projections along the *Z*-axis of at least five images at optimal thickness from each slide were obtained using a FLUOVIEW (version 4.02, Olympus). Images were processed in brightness and contrast using an Adobe Photoshop Elements14 (Adobe Systems, San Jose, CA).

In some experiments, secondary antibodies conjugated with horseradish-peroxidase (Vector Laboratories, Burlingame, CA) were used and immune complexes were detected with either a Vectastain Elite ABC kit (Vector Laboratories) or an EnVision kit (DAKO, Glostrup, Denmark) using 3,3′-diaminobenzidine (DAB) as a substrate. Images were acquired using a light microscope (IX71, Olympus) equipped with a UPlanFLN 4×/NA 0.13 objective (Olympus), UPlanFLN 10×/NA 0.3 objective (Olympus), or LUCPlanFLN 40×/NA 0.6 objective (Olympus) at a resolution of 1360 × 1024 pixels with a field of view of 4.4 × 3.3 mm, 1.8 × 1.3 mm, or 438.6 × 330.2 μm, respectively. Images were captured using a digital camera (DP72, Olympus) and a cellSens Standard (version 1.6, Olympus). The brightness and contrast of the images were adjusted in an Adobe Photoshop Elements14 (Adobe Systems).

Primary antibodies used are as follows: rabbit polyclonal anti-GPR120 antibody (dilution 1:900, #LS-A2003, LifeSpan BioSciences, Inc., Seattle, WA), mouse monoclonal anti-CD31 antibody (1:100, #M0823, DAKO), mouse monoclonal anti-smooth muscle α-actin (SMA) antibody (1:200, #M0851, DAKO), and mouse monoclonal anti-CD68 antibody (1:100, #M0814, DAKO).

The secondary antibodies used are as follows: Alexa Fluor 488-conjugated donkey anti-rabbit IgG (H + L) antibody (1:100, #A21206, Invitrogen, Carlsbad, CA) and Alexa Fluor 594-conjugated donkey anti-mouse IgG (H + L) antibody (1:100, #A21203, Invitrogen).

### Rodent IA models and histological analysis of induced IA

Male Sprague–Dawley rats were purchased from Japan SLC (*n* = 84, Shizuoka, Japan). Animals were maintained on a light/dark cycle of 12 h/12 h, and had a free access to chow and water.

A total of 24 rats were used to examine the pharmacokinetics of eicosapentaenoic acid (EPA). The whole blood was transcardially collected from four rats before EPA administration under intraperitoneal injection of a lethal dose of pentobarbital sodium (200 mg/kg). The remaining 20 rats were randomly divided into the two groups; vehicle-treated group and the EPA-treated group. Ten rats in each group were administrated 1000 mg/kg EPA or olive oil as a vehicle. The whole blood was then collected transcardially at 1 h (*n* = 3), 6 h (*n* = 3), or 24 h (*n* = 4) after the administration.

To induce IA, under general anesthesia by intraperitoneal injection of pentobarbital sodium (50 mg/kg), 7-week-old male rats were subjected to ligation of the left carotid artery and hypervolemia achieved by the combination of a high salt diet and ligation of the left renal artery. Immediately after above surgical manipulations, the chow was completely switched from a normal to the special one containing 8% sodium chloride and 0.12% 3-aminopropionitrile (Tokyo Chemical Industry, Tokyo, Japan), an inhibitor of lysyl oxidase that catalyzes the cross-linking of collagen and elastin. 3-Aminopropionitrile was added in a chow to reduce the stiffness of arterial walls and thus facilitate degenerative changes of the media. The special chow was refilled twice a week, and all rats kept free access to water and the special chow after surgical manipulations during the observation period. A total of 60 rats were subjected to surgical manipulations and 30 rats among them were used for examining the effect of EPA on IAs. Another 30 rats were used to determine the intake of EPA in the plasma. These 30 rats were randomly allocated into three groups: the olive oil-administered group (vehicle-treated group, *n* = 10), the 100 mg/kg/day EPA-administered group (*n* = 10), or the 1000 mg/kg/day EPA-administered group (*n* = 10). Dead animals during the observation period were excluded from the analysis. Blood pressure was measured by the tail-cuff method on the 11th day after surgical manipulations. The rats were placed in a cylindrical warmer (#THC-31, Softron, Tokyo, Japan) and kept at 37 °C during the measurement. Blood pressure were then measured using an automatic measurement device (#BP-98A, Softron) without any anesthesia and its value was defined as the average of three consecutive measurements. Body weight was measured twice; before surgical manipulations and on the day of sacrifice. At times indicated in the corresponding figure legends or results after above surgical manipulations, animals were deeply anesthetized by intraperitoneal injection of pentobarbital sodium (200 mg/kg), and transcardially perfused with 4% paraformaldehyde solution. The bifurcation site of anterior cerebral artery (ACA)-olfactory artery (OA) including the induced IA lesion was then stripped, and serial frozen sections were made. Histopathological examination to evaluate IAs was done after Elastica van Gieson staining which visualizes internal elastic lamina (IEL) and induced IAs were histologically defined as a lesion with the disruption of IEL [8, 13]. The area of IA was measured using ImageJ software (version 1.51s, https://imagej.nih.gov/ij/index.html) [14] using the section including the maximum diameter of the lesions.

### EPA

EPA (Epadel (ethyl icosapentate), Mochida Pharmaceutical Co., LTD., Tokyo, Japan) was commercially available and we thus purchased this drug. Immediately before administration, EPA was extracted from Epadel capsule and then diluted with olive oil to the concentration of 25 mg/ml or 250 mg/ml. Then, 4 ml/kg of EPA solution was orally administered by gavage. The dose of EPA was determined by referencing the previous reports in a rat model [15–17]. For the measurement of plasma concentration of EPA, docosahexaenoic acid (DHA), and also arachidonic acid (AA), arterial blood was transcardially collected with an 18-gauge needle at 24 h after the last administration of EPA or vehicle after intraperitoneal injection of a lethal dose of pentobarbital sodium (200 mg/kg). The collected blood (5–8 ml per animal) was heparinized and the plasma was then collected. The plasma samples were stored at − 80 °C until measurement. The plasma concentration of EPA, DHA, and AA was measured by gas chromatography at SRL Inc. (Tokyo, Japan).

### Immunohistochemistry

At the indicated period after surgical manipulations, 5-μm-thick frozen sections were prepared from dissected IA lesions as described above. After blocking with 3% donkey serum (Jackson ImmunoResearch), slices were incubated with primary antibodies overnight at 4 °C in a humidified chamber, followed by incubation with secondary antibodies conjugated with a fluorescence dye (Jackson ImmunoResearch) for additional 1 h at room temperature in a humidified chamber. The slices were then mounted with by a ProLongR Gold antifade reagent with DAPI (4′,6-diamidino-2-phenylindole dihydrochloride) (Invitrogen), a fluorescence dye that binds to double-stranded DNA and then visualizes nuclei. Finally, fluorescent images were acquired on a confocal fluorescence microscope system (FV1000, Olympus). Scanning was performed using a UPLSAPO 40×/NA 0.9 objective (Olympus) or 60×/NA 1.35 oil immersion objective (Olympus) at a resolution of 1024 × 1024 pixels with a field of view of 317 μm or 211 μm square, respectively. Maximal intensity projections along the *Z*-axis of at least five images at optimal thickness from each slide were obtained using a FLUOVIEW (version 4.02, Olympus). Images were processed in brightness and contrast using an Adobe Photoshop Elements14 (Adobe Systems).

The primary antibodies used are as follows: Cy3-conjugated mouse monoclonal anti-smooth muscle α-actin antibody (1:200, #6198-2ML, Sigma Aldrich, St. Louis, MO), mouse monoclonal anti-CD68 antibody (1:100, #31630, Abcam, Cambridge, UK), and rabbit polyclonal anti-C-C motif chemokine 2 (CCL2) antibody (1:100, #ab9779, Abcam).

The secondary antibodies used are as follows: Alexa Fluor 488-conjugated donkey anti-mouse IgG (H + L) antibody (1:100, #A21202, Invitrogen) and Alexa Fluor 488-conjugated donkey anti-rabbit IgG (H + L) antibody (1:100, #A21206, Invitrogen).

### Macrophage count

Macrophage was defined as the cell positive for CD68 in immunohistochemistry. The number of infiltrated macrophages in each lesion was calculated as a cell count present around the dome of induced aneurysms. Independent two researchers (Y.A. and I.O.) who did not know the group allocation counted the number of CD68-positive cells in each slide and the results were compared. When disagreed, the researchers checked and discussed the image together, and determined the number of positive cells.

### Plasma concentration of Resolvin E1

Plasma was prepared from rats administered for 12 days with 1000 mg/kg/day of EPA. The plasma was collected at 24 h after the last administration of EPA as described above. And the concentration of Resolvin E1 was measured by a Rat Resolvin E1 ELISA Kit (#MBS2601346, MyBioSource, Inc., San Diego, CA) according to the manufacturer’s instructions.

### Cell culture and treatment with compounds targeting GPR120

The Raw264.7 cell line (ATCC, Manassas, VA), a mouse monocyte/macrophage cell line, was used due to endogenous expression of functional GPR120 [11]. Cells were cultured in Dulbecco’s modified Eagle medium (DMEM) (FUJIFILM Wako Pure Chemical Corporation, Osaka, Japan) supplemented with 10% fetal bovine serum. In some experiments using EPA, DMEM without phenol-red (FUJIFILM Wako Pure Chemical Corporation) was used. Cells were pre-treated with GPR120 agonists, EPA (Mochida Pharmaceutical Co., Ltd.) or TUG-891 (Cayman Chemical, Ann Arbor, MI), before stimulation with lipopolysaccharide (LPS) (Lot. 123M4052V, # L2654, Sigma Aldrich).

### Western blot analysis

Whole cell lysate was prepared by a RIPA buffer (Sigma Aldrich) supplemented with proteinase and phosphatase inhibitors (Roche, Indianapolis, IN). Protein concentration was, then, determined by a bicinchoninic acid (BCA) method (Pierce BCA Protein Assay Kit, Thermo Scientific, Waltham, MA). After sodium dodecyl sulfate-poly-acrylamide gel electrophoresis (SDS-PAGE), separated proteins were transferred to a PDVF membrane (Hybond-P, GE healthcare, Buckinghamshire, UK) and blocked with an ECL plus blocking agent (GE healthcare). The membranes were incubated with primary antibodies followed by incubation with an anti-IgG antibody conjugated with horseradish peroxidase (GE healthcare). Finally, the signal was detected by a chemiluminescent reagent (ECL Prime Western Blotting Detection System, GE healthcare). α-Tubulin was served as an internal control.

Antibodies used in Western blot analysis are as follows: rabbit monoclonal anti-p65 antibody (Cell Signaling Technology, Danvers, MA), rabbit monoclonal anti-phospho NF-κB p65 (Ser536) antibody (Cell Signaling Technology), and mouse monoclonal anti-α-tubulin antibody (Sigma Aldrich).

### Quantitative real time-PCR analysis

RNA purification from cultured cells and reverse transcription were done using a RNeasy Plus Mini Kit (QIAGEN, Hilden, Germany) and a High-capacity cDNA Reverse Transcription Kit (Life Technologies Corporation, Carlsbad, CA) according to manufacturers’ instructions. For the quantification of gene expression, RT-PCR was performed on a Real Time System a LightCycler 480 (Roche) with a TB Green Premix Ex Taq II (TAKARA BIO INC., Shiga, Japan) using the expression of α-actin (*Actb*) as an internal control. For quantification, the second derivative maximum method was used for crossing point determination.

Primer sets used in the present experiment are as follows: forward 5′-GTCTCTGCCGCCCTTCTGTG-3′ and reverse 5′-AGGTGACTGGGGCATTGATTG-3′ for *Ccl2* which encodes monocyte chemoattractant protein 1 (MCP-1), and 5′-ACGACCAGAGGCATACAGGGA-3′ and 5′-CCCTAAGGCCAACCGTGAAA-3′ for *Actb*.

### Statistics

Data are shown as mean ± SD or by box-and-whisker plots. Differences between the two groups were examined using a non-parametric Mann-Whitney test by a JMP Pro 13 (SAS Institute Inc., Cary, NC). Statistical comparisons between more than two groups were conducted using a Kruskal-Wallis test followed by the post-hoc Steel-Dwass test by a JMP Pro 13 (SAS Institute Inc.). We used above non-parametric tests in the present study because the sample size was not enough to assess whether the data sets were normally distributed or not by a Shapiro-Wilk test. The Jonckheere-Terpstra test was performed to confirm a dose-response relationship using an EZR software (Saitama Medical Center, Jichi Medical University, Saitama, Japan, http://www.jichi.ac.jp/saitama-sct/SaitamaHP.files/statmedEN.html) [18]. A *p* value smaller than 0.05 was defined as statistically significant.

## Results

### Over-expression of *GPR120* in human IA walls revealed by RNA sequencing analysis and protein expression of GPR120 in human IA lesions

**Fig. 1:**
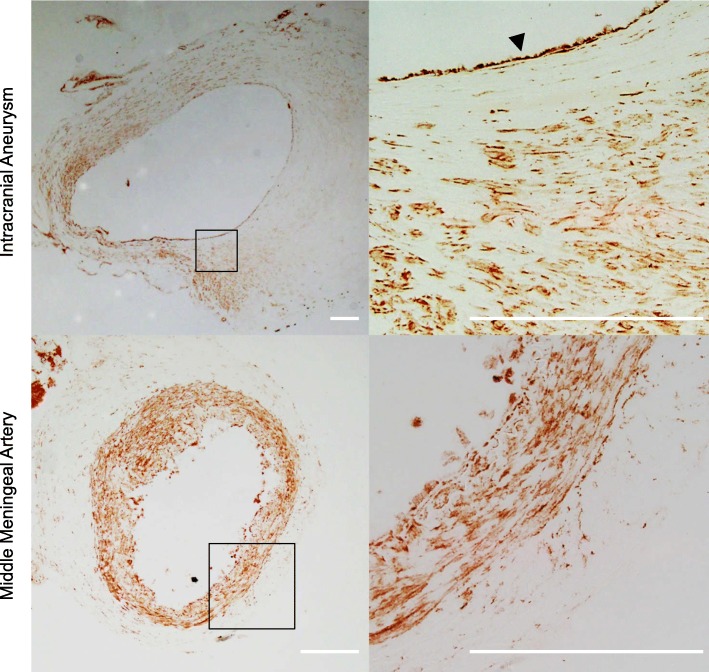
Expression of GPR120 in human IA lesion and the control arterial wall. The representative images of immunostaining of specimens from human IA lesion (the upper panels) and the control arterial wall (middle meningeal artery, the lower panels) for GPR120 are shown. The magnified images corresponding to the squares in the left panels are also shown on a right. Bar; 200 μm. The arrow head indicates the endothelial cells where GPR120 expression is induced

**Fig. 2:**
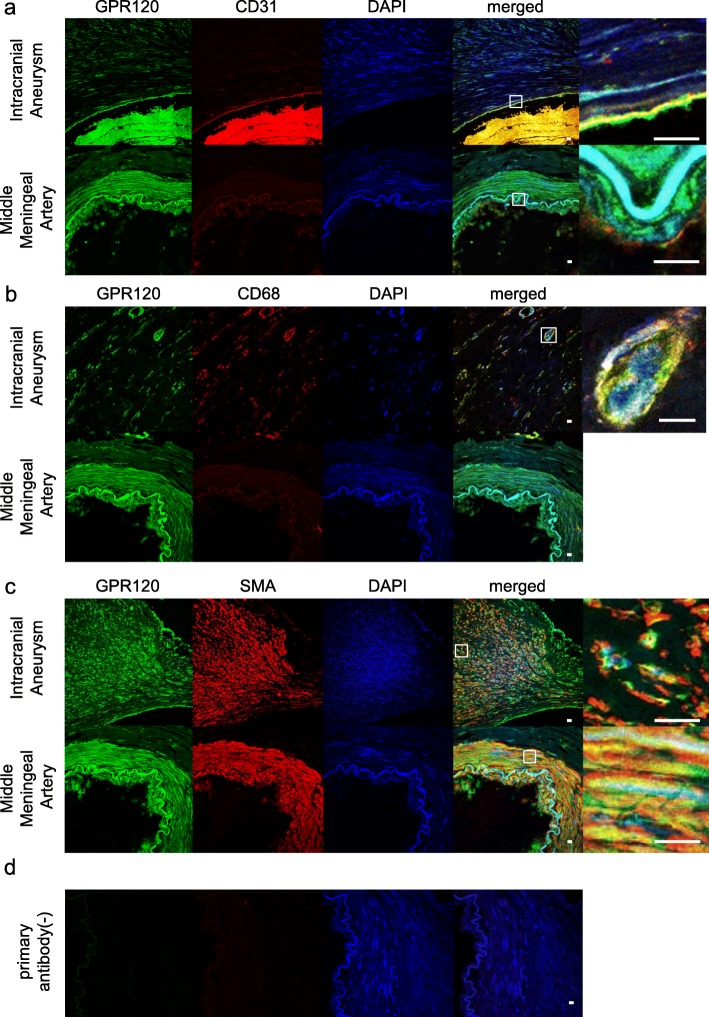
Induction of GPR120 expression in endothelial cells and macrophages of human IA lesions. The representative images of immunostaining of specimens from human IA lesion (the upper panels) and the control arterial wall (middle meningeal artery, the lower panels) for GPR120 (green), CD31, a marker for endothelial cells (red in **a**), CD68, a marker for macrophages (red in **b**) or smooth muscle α-actin (SMA), a marker for smooth muscle cells (red in **c**), nuclear staining by DAPI (blue) and merged images are shown. The magnified images corresponding to the squares in the left panels are also shown on a right. In **d**, the image of immunostaining without a primary antibody is shown as a negative control study. Bar; 10 μm. Note the induction of GPR120 in CD31-positive endothelial cells and CD68-positive macrophages while expression in smooth muscle cells is constitutive

We picked up GPR120 (free fatty acid receptor 4 (FFAR4)) as a putative therapeutic target from GPCRs whose expression was significantly upregulated in human unruptured IA lesions (*n* = 4) compared with those in control arterial walls (*n* = 3) (*p* = 7.95E^−10^) from previously obtained comprehensive gene expression profile data by RNA-sequencing analysis [10] (ID number #PRJNA553307 at BioProject database (http://www.ncbi.nlm.nih.gov/bioproject)). To confirm whether over-expression of *GPR120* is indeed reflected in protein expression, we performed immunohistochemical analysis using both human IA specimens (*n* = 4) and control arterial walls (*n* = 3). In control arterial walls, signals for GPR120 in immunostaining were detected throughout the media and sparsely in the adventitia but not in the intima (Fig. 1 and Additional file 1: fig. S1). In IA lesions, signals for GPR120 in the media became sparse, presumably due to loss of medial smooth muscle cells, but signals in the intima was induced (Fig. 1 and Additional file 1: fig. S1). Positive signals for GPR120 staining were co-localized with the marker of smooth muscle cells, SMA, both in IA lesions and control arterial walls (Fig. 2). In addition, other positive signals for GPR120 staining observed in IA walls were co-localized with the marker of macrophages, CD68, in the adventitia or the marker of endothelial cells, CD31, in the intima (Fig. 2), suggesting the expression of GPR120 in macrophages and the induction of this receptor in endothelial cells during the progression of the disease.

### Effect of a ligand of GPR120, EPA, on the progression of IAs induced in a rat model

**Fig. 3:**
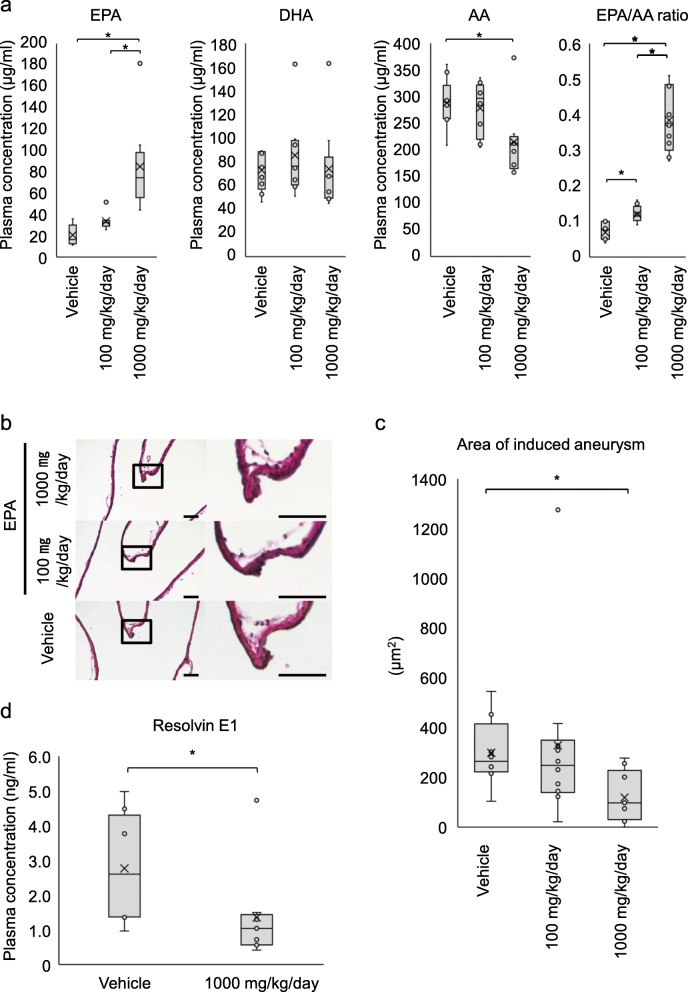
Inhibitory effect of EPA on the size of intracranial aneurysms in a rat model. **a** Plasma concentration of EPA, docosahexaenoic acid (DHA), and arachidonic acid (AA) in rats orally administered EPA. Rats were orally administered EPA (vehicle, *n* = 9, 100 mg/kg/day, *n* = 8, 1000 mg/kg/day, *n* = 9) once a day and, on the 5th day, plasma concentration of EPA, DHA, and AA was measured and the ratio of EPA over AA was also calculated. Data represents box-and-whisker plots. Statistical analysis was done by a Kruskal-Wallis test. *p < 0.05. **b**, **c** Effect of EPA on the size of induced aneurysms. EPA was administered in a rat model subjected to aneurysm induction once a day for 12 days and the size of induced aneurysms at right anterior cerebral-olfactory artery bifurcation was measured after Elastica van Gieson staining (vehicle, *n* = 8, 100 mg/kg/day, *n* = 10, 1000 mg/kg/day, *n* = 9). The representative microscopic images of induced aneurysms with Elastica van Gieson staining in each group are shown in **b**. Bar; 50 μm. Data represents box-and-whisker plots (**c**). Statistical analysis was done by a Kruskal-Wallis test. *p < 0.05. **d** Plasma concentration of Resolvin E1 in rats administered EPA. Rats were orally administered EPA once a day and, after 12 days, plasma concentration of Resolvin E1 was measured by ELISA (vehicle, *n* = 8, 1000 mg/kg/day, *n* = 9). Data represents box-and-whisker plots. Statistical analysis was done by a Mann-Whitney test. *p < 0.05

We selected EPA as an agonist for GPR120 [11, 19] because EPA is the already-approved drug for a clinical usage. We first examined the temporal change of the plasma concentration of EPA after the oral administration of this compound to confirm that EPA orally administrated was certainly taken into the plasma in rats. The plasma concentration of EPA before administration was 32.8 ± 2.7 μg/ml (*n* = 4). The plasma concentration of EPA was certainly and significantly increased by the oral administration of EPA (1000 mg/kg) compared with the concentration in vehicle-treated group (1 h, 38.2 ± 2.0 vs. 139.0 ± 26.1 μg/ml, vehicle-treated group (*n* = 3) vs. 1000 mg/kg/day-administered group (*n* = 3), *p* = 0.049; 6 h, 28.4 ± 1.4 vs. 241.0 ± 66.2 μg/ml, vehicle-treated group (*n* = 3) vs. 1000 mg/kg/day-administered group (*n* = 3), *p* = 0.049; 24 h, 34.7 ± 6.6 vs. 114.8 ± 12.4 μg/ml, vehicle-treated group (*n* = 4) vs. 1000 mg/kg/day-administered group (*n* = 4), *p* = 0.020) (Additional file 1: fig. S2 left). Because DHA is a derivative of EPA [20, 21], the plasma concentration of DHA was also measured to rule out the effect of DHA when EPA was administered. We then found that the plasma concentration of DHA was not increased by the oral administration of EPA (Additional file 1: fig. S2 right). We thereby administered 100 mg/kg/day or 1000 mg/kg/day EPA to the rat model of IAs and again examined the plasma concentration of EPA or related factors on the 5th day after surgical manipulations to induce IAs. We confirmed the increase of EPA (vehicle-treated group, 16.3 [12.3–29.9] μg/ml, *n* = 9; 100 mg/kg/day-administered group, 32.2 [28.8–34.2] μg/ml, *n* = 8; 1000 mg/kg/day-administered group, 74.3 [55.6–97.9] μg/ml (median [interquartile range]), *n* = 9; vehicle-treated group (*n* = 9) vs. 1000 mg/kg/day-administered group (*n* = 9), *p* = 0.0012, 100 mg/kg/day-administered group (*n* = 8) vs. 1000 mg/kg/day-administered group (*n* = 9), *p* = 0.0026) and also the elevation of EPA/AA ratio in a dose-dependent manner (*p* = 3.80E^-7^, the Jonckheere-Terpstra test) (Fig. 3a). The body weight was significantly increased during the observation period in all groups examined (vehicle-treated group, 234.0 ± 5.2 vs. 280.4 ± 16.1 g, pre-treatment vs. post-treatment, *n* = 9, *p* = 0.0039; 100 mg/kg/day-administered group, 226.6 ± 6.8 vs. 269.8 ± 9.6 g, *n* = 10, *p* =0.002; 1000 mg/kg/day-administered group, 233.8 ± 4.1 vs. 273.3 ± 17.0 g, *n* = 9, *p* = 0.0039). Also the administration of EPA did not influence the systolic blood pressure as well (Additional file 1: fig. S3). On the 12th day after IA induction, all rats examined developed IAs characterized by the disruption of an internal elastic lamina in Elastica van Gieson staining (Fig. 3b). Three of 30 rats (10%) surgically manipulated died on the 3rd, 7th, and 11th day after the manipulations, and these animals were excluded from the analyses (vehicle-treated group, *n* = 2, 1000 mg/kg/day-administered group, *n* = 1). Thereby, the mortality rate during the observation period was 2/10 (20%) in vehicle-treated group, 0/10 (0%) in 100 mg/kg/day-administered group, and 1/10 (10%) in 1000 mg/kg/day-administered group. Though the incidence of IAs was not influenced by the administration of EPA, the size of the lesions was significantly suppressed in a dose-dependent manner (*p* = 0.012, the Jonckheere-Terpstra test) (vehicle-treated group, 264.3 [222.1–415.3] μm^2^, *n* = 8; 100 mg/kg/day-administered group, 247.9 [138.9–349.5] μm^2^, *n* = 10; 1000 mg/kg/day-administered group, 97.7 [29.5–228.3] μm^2^, *n* = 9; vehicle-treated group vs. 1000 mg/kg/day-administered group, *p* = 0.037) (Fig. 3b, c).

We used EPA as an agonist for GPR120. However, as well-known, EPA exerts it action not only via GPR120 as an agonist but also via the formation of the metabolite, Resolvin, with a potent anti-inflammatory effect [22–27]. We therefore examined the concentration of Resolvin E1 in the plasma collected from EPA-administered animals. The plasma concentration of Resolvin E1 in animals treated with 1000 mg/kg/day of EPA for 12 days was rather significantly lower than that in the vehicle-treated group (2.60 [1.36–4.30] vs. 1.03 [0.56–1.43] ng/ml, vehicle-treated group (*n* = 8) vs. 1000 mg/kg/day-administered group (*n* = 9), *p* = 0.048) (Fig. 3d), supporting that the suppressive effect of EPA on the progression of IAs was via GFP120.

### Effect of EPA on degenerative changes of media and inflammatory responses in IA lesions

**Fig. 4:**
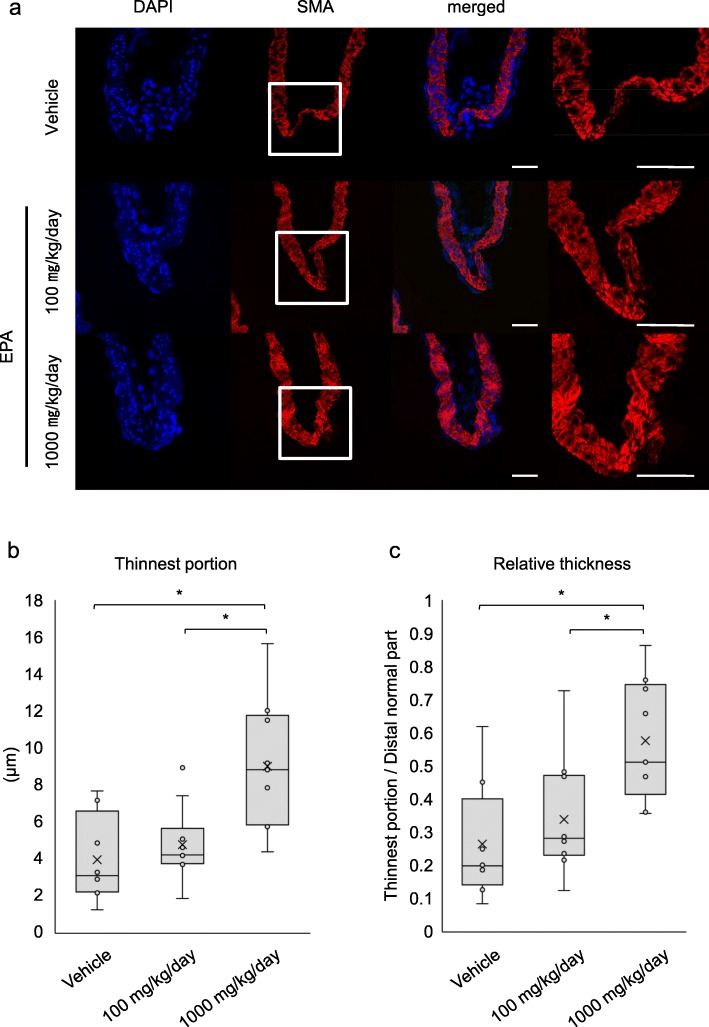
Inhibitory effect of EPA on the thinning of medial smooth muscle cell layer in lesions. **a**–**c** Inhibitory effect of EPA on the thinning of medial smooth muscle cell layer. Medial smooth muscle cells in IA lesions from rats treated with vehicle or EPA (100 or 1000 mg/kg/day) for 12 days were visualized by immunostaining for smooth muscle α-actin (SMA), a marker for smooth muscle cells. The representative images from immunohistochemistry for SMA (red), nuclear staining by DAPI (blue) and merged images are shown (**a**). The magnified images corresponding to the squares are shown on a right (**a**). Bar; 50 μm. The thickness of the thinnest portion in the media of IA lesions (**b**) and the relative thickness over that of the distal normal part (**c**) were calculated (vehicle, *n* = 8, 100 mg/kg/day, *n* = 10, 1000 mg/kg/day, *n* = 9). Data represents box-and-whisker plots. Statistical analysis was done by a Kruskal-Wallis test. *p < 0.05

We, next, examined the effect of EPA on degenerative changes of media and inflammatory responses in IA lesions, one of the hallmarks reflecting the disease progression [28, 29]. The thinning of medial smooth muscle cell layer in lesions visualized by immunohistochemistry for a smooth muscle cell marker, SMA, was significantly suppressed by the administration of EPA (thickness of the thinnest portion in lesions; 3.10 [2.23–6.60] vs. 8.83 [5.85–11.7] μm, vehicle-treated group (*n* = 8) vs. 1000 mg/kg/day-administered group (*n* = 9), *p* = 0.0168, 4.23 [3.76–5.67] vs. 8.83 [5.85–11.7] μm, 100 mg/kg/day-administered group (*n* = 10) vs. 1000 mg/kg/day-administered group (*n* = 9), *p* = 0.0134, relative thickness; 0.19 [0.14–0.40] vs. 0.51 [0.41–0.74], vehicle-treated group (*n* = 8) vs. 1000 mg/kg/day-administered group (*n* = 9), 0.28 [0.23–0.47] vs. 0.51 [0.41–0.74], 100 mg/kg/day-administered group (*n* = 10) vs. 1000 mg/kg/day-administered group (*n* = 9), *p* = 0.027) (Fig. 4).

**Fig. 5:**
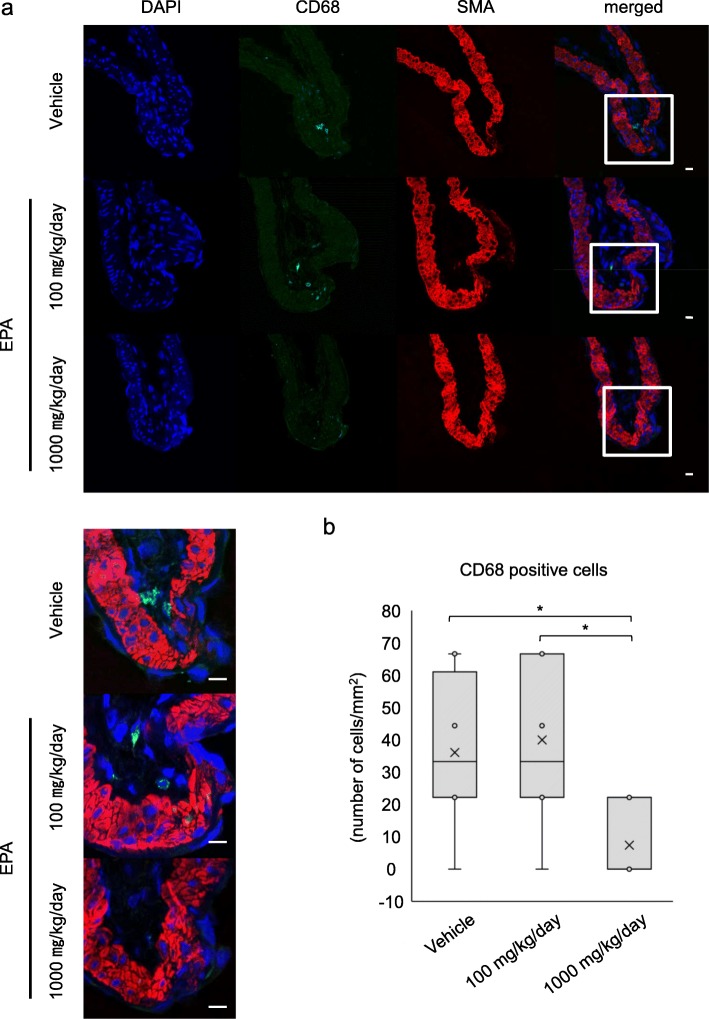
Inhibitory effect of EPA on the infiltration of macrophages in lesions. **a**, **b** Inhibitory effect of EPA on the infiltration of macrophages in lesions. Macrophages infiltrating in IA lesions from rats treated with vehicle or EPA (100 or 1000 mg/kg/day) for 12 days were visualized by immunostaining for CD68, a marker for macrophages. The representative images from immunohistochemistry for CD68 (green) or SMA, a marker for smooth muscle cells (red), nuclear staining by DAPI (blue) and merged images are shown (**a**). The magnified images corresponding to the squares are also shown in the lower panels (**a**). Bar; 10 μm. The number of infiltrating macrophages in IA lesions was calculated (vehicle, *n* = 8, 100 mg/kg/day, *n* = 10, 1000 mg/kg/day, *n* = 9). Data represents box-and-whisker plots (**b**). Statistical analysis was done by a Kruskal-Wallis test. *p < 0.05

We then assessed the infiltration of macrophages which play the crucial role in the pathogenesis of IAs through mediating chronic inflammation [8, 9, 13, 28, 30–33]. The infiltration of CD68-positive macrophages into IA lesions was significantly suppressed by the administration of EPA (33.2 [22.1–61.0] vs. 0.0 [0.0–22.1] cells/mm^2^, vehicle-treated group (*n* = 8) vs. 1000 mg/kg/day-administered group (*n* = 9), *p* = 0.028, 33.2 [22.1–66.5] vs. 0.0 [0.0–22.1] cells/mm^2^, 100 mg/kg/day-administered group (*n* = 10) vs. 1000 mg/kg/day-administered group (*n* = 9), *p* = 0.013) (Fig. 5).

### Anti-inflammatory effect of EPA in RAW264.7 cell line and in a rat model

**Fig. 6:**
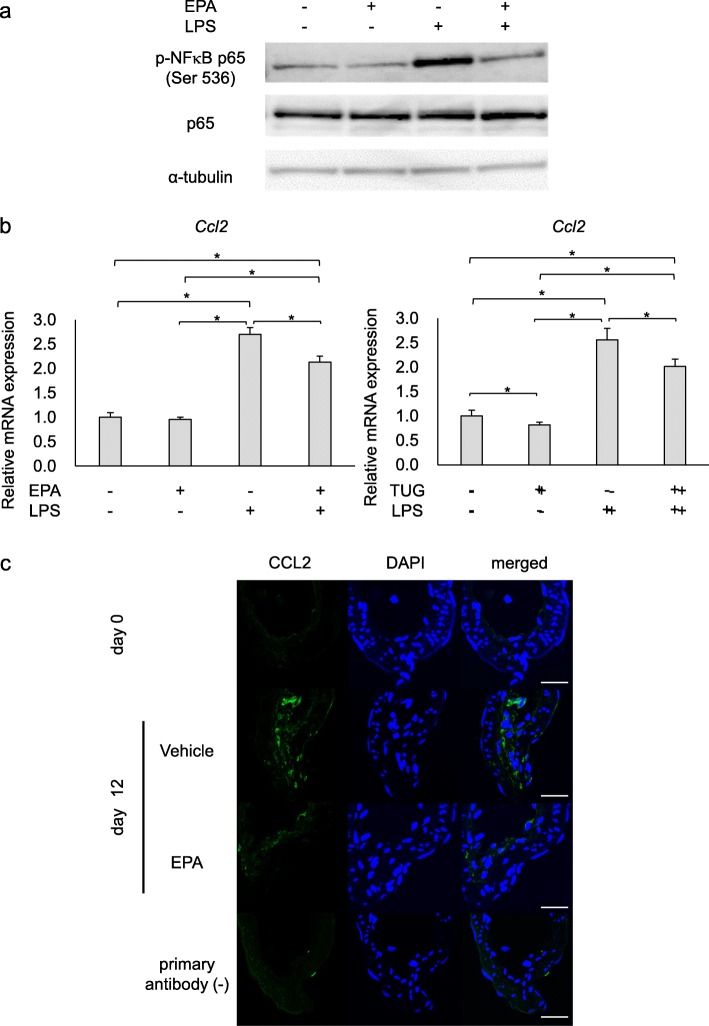
Suppression of NF-κB activation and CCL2 expression by EPA treatment in vitro. **a** Suppression of LPS-induced NF-κB activation by the pre-treatment with EPA. RAW264.7 cells were treated with EPA (300 μM) for 60 min and then stimulated with LPS (3.3 μg/ml) for additional 10 min. NF-κB activation was then assessed by Western blot analysis using the whole cell lysate. The representative images of Western blot analyses from two independent experiments for phosphorylated form of NF-κB p65 subunit (Ser536), NF-κB p65 subunit or α-tubulin served as an internal control are shown. **b** Suppression of LPS-induced *Ccl2* expression by the pre-treatment with EPA. RAW264.7 cells were treated with EPA (300 μM, *n* = 6) or an agonist for GPR120, TUG-891 (3 μM, *n* = 7), for 60 min and then stimulated with LPS (3.3 μg/ml) for additional 60 min. *Ccl2* expression was then assess by RT-PCR analyses. Data represents mean ± SD. Statistical analysis was done by a Kruskal-Wallis test. *p < 0.05. **c** Suppression of CCL2 expression in IA lesions by EPA in rats. EPA (1000 mg/kg/day) was administered in a rat model subjected to aneurysm induction once a day for 12 days and expression of CCL2 in IA lesions was then assessed by immunohistochemistry. The representative images from immunohistochemistry for CCL2 (green), nuclear staining by DAPI (blue), and merged images are shown. The image from immunostaining without a primary antibody for CCL2 is also shown as a negative control study in the lowest panel. Bar; 20 μm

Referencing the previous report demonstrating endogenous expression of GPR120 in RAW264.7 cell line [11] and considering the crucial role of macrophages in the pathogenesis [9, 30, 31], we used this line to examine the effect of EPA on inflammatory responses observed in in vivo (Fig. 5). We first examined whether EPA indeed could modulate LPS-induced inflammation via GPR120. Referencing the previous study that EPA suppresses activation of NF-κB via GPR120 [11, 34], we examined the effect of exogenous EPA on the phosphorylation of NF-κB p65 subunit at Serine 536 residue by Western blot analysis. Addition of EPA inhibited LPS-induced phosphorylation of NF-κB p65 subunit (Ser536) as expected (Fig. 6a and Additional file 1: fig. S4). We next assessed *Ccl2* expression in these cells by RT-PCR analysis considering the pivotal role of macrophages in the pathogenesis [30, 33, 35] and also the inhibition of macrophage infiltration in lesions by EPA (Fig. 5). EPA partially but significantly suppressed LPS-induced *Ccl2* expression consistently with the result in NF-κB activation (Fig. 6b). Importantly, a selective GPR120 agonist, TUG-891 [36, 37], reproduced the effect of EPA on LPS-induced *Ccl2* expression (Fig. 6b). Since induction of MCP-1 encoded by *Ccl2* leads to the formation of a self-amplifying loop among macrophages to exacerbate inflammatory responses by recruiting more macrophages to the inflammatory microenvironment, which in turn promotes the progression of IAs [9], we next examined whether EPA indeed inhibits MCP-1 expression in vivo in a rat model. As a result, CCL2 expression was induced in IA lesions especially at the adventitia and its expression was remarkably suppressed in IA lesions from EPA-administered rats (Fig. 6c), supporting the in vivo relevance of the in vitro study (Fig. 6a, b).

The absolute values of each parameter are listed in the Additional files 2, 3, 4 and 5: Table S1-S4.

## Discussion

In the present study, we used an agonist targeting GPR120, EPA, and suggested the suppressive role of this receptor signaling in the progression of IAs induced in rats (Additional file 1: fig. S5). However, EPA has various sites of action on inflammation beyond being a GPR120 agonist, thus, we failed to provide experimental evidence that EPA exerts its effect on the progression of the disease uniquely via GPR120 because of the lack of a rat line deficient in this receptor. Although in vitro studies supported the anti-inflammatory action of EPA at least partially through GPR120 in macrophages and previous studies consistently demonstrated the crucial role of GPR120 signaling in the EPA-mediated suppression of inflammatory responses [11, 34]. The precise contribution of GPR120 signaling to the pathogenesis remains to be carefully investigated. One of the possible mechanisms underlying EPA-mediated suppression of the disease progression may incorporate metabolites such as resolvins [22–25]. As the plasma concentration of Resolvin E1 was not increased by the administration of EPA in the present study (Fig. 3d), we assumed that EPA directly exerts anti-inflammatory action in IA lesions. Another possibility is that EPA is integrated into the plasma membrane and affects the fluidity of the lipid bilayer presumably leading to changes in shear stress-sensing. To further clarify the role of GPR120 signaling in the progression of the disease, the establishment of a rat line deficient in GPR120 is required.

We have demonstrated the suppressive effect of hydroxymethylglutaryl-coenzyme A (HMG-CoA) reductase inhibitors on the rupture of human IAs [38], and there are also many reports describing similar suppressive effects of non-steroid anti-inflammatory drugs (NSAIDs) [39, 40]. These two classes of drugs both presumably exert an inhibitory effect on the rupture of IAs through their potent anti-inflammatory effect, the anti-NF-κB effect of statins [41, 42] and the suppression of pro-inflammatory prostaglandin synthesis [43, 44]. The development of a drug therapy to prevent the rupture of IAs by targeting inflammation is thus quite promising. Each group of drugs has, however, adverse effects that requires scrupulous attention when being used as long-term-administered preemptive drugs. HMG-CoA reductase inhibitors decrease serum cholesterol level below the normal limit even healthy individuals but a safety of long time-exposure of such a low serum cholesterol level has not yet been elucidated, making application of this class of drugs for preventing SAH difficult. In the case of NSAIDs, gastrointestinal toxicity or a bleeding tendency due to interference with the physiological functions of prostanoids, including prostaglandins, also makes the development of this class of drugs as therapeutic agents to prevent the onset of SAH quite challenging. With respect to the concern about the bleeding tendency caused by NSAIDs and an application for the treatment of unruptured IAs, there exist controversial reports, with some of them demonstrating a preventive [39, 40] while others, an exacerbating effect [45], toward SAH. As EPA does not have adverse effects on the disease state or performance status, EPA could serve as a reasonable candidate for the treatment of IAs.

## Conclusions

Considering the devastating outcome of SAH due to rupture of IA and the lack of the medical therapy to prevent the progression or rupture of the lesions, the development of the novel medical therapy is mandatory for social health. In the present study, we have identified GPR120 as a GPCR over-expressed in lesions from the comprehensive gene expression profile analyses data using human specimen and also clarified the preventive effect of a GPR120 agonist, EPA, on the progression of the disease using a rat model of IAs. The results of the present study have thus implied the potential of EPA as a drug to prevent the progression of IAs.

## Supplementary information

**Additional file 1: Fig. S1.** GPR120 expression in human IA lesions. The representative image of immunostaining for GPR120 using human IA specimen is shown in the left panel. The image from immunostaining without a primary antibody for GPR120 is shown as a negative control study in the right panel. The image in the left panel is the same one used in Fig. 1. Bar; 200 μm. **Fig. S2.** Temporal change of the concentration of EPA and DHA in plasma of rats orally administered EPA. EPA (1000 mg/kg) was orally administered to rats and the concentration of EPA and DHA in plasma was measured at 1 h (n=3), 6 h (n=3) or 24 h (n=4) after the administration. Data represents mean ± SD. Statistical analysis was done by a Mann-Whitney test. *; p<0.05. Note that the plasma concentration of DHA was not increased by the administration of EPA. **Fig. S3.** Body weight and systemic blood pressure of rats treated with EPA. Rats were subjected to surgical manipulations to induce IAs and then given vehicle or EPA (100 or 1000 mg/kg/day) for 12 days. Body weight (the left panel) and systemic blood pressure (the right panel) were then measured (vehicle, n=9, 100 mg/kg/day, n=10, 1000 mg/kg/day, n=9). Data represents mean ± SD. Statistical analysis was done by a Kruskal-Wallis test. **Fig. S4.** Full-scanned images of western blot analysis in Fig. 6a. RAW264.7 cells were treated with EPA (300 μM) for 60 min and then stimulated with LPS (3.3 μg/ml) for additional 10 min. NF-κB activation was then assessed by western blot analysis using the whole cell lysate. The whole membranes of the western blot analysis presented in Fig. 6a are shown. Protein molecular weight markers are also displayed on both sides. **Fig. S5.** The graphical abstract of the suppressive effects of EPA on the progression of IAs. The one of the major mechanisms underlying the suppressive effect of EPA on the progression of IAs is the inhibition of inflammation by macrophages through interfering NF-κB activation via GPR120. Note the interruption of the MCP-1-mediated self-amplification loop among macrophages by EPA. In addition, anti-inflammatory effect of EPA via GPR120 expressed on endothelial cells and the disturbance of wall shear stress-sensing due to the integration of EPA into lipid bilayer in this cell type may also suppress the progression of IAs.

**Additional file 2: Table S1.** The raw data corresponding to Fig. 3a.

**Additional file 3: Table S2.** The raw data corresponding to Fig. 3b-d, Fig. 4, Fig. 5, and fig. S3.

**Additional file 4: Table S3.** The raw data corresponding to fig. S2.

**Additional file 5: Table S4.** The raw data corresponding to fig. S3.

## Data Availability

The datasets used and/or analyzed during the current study are available from the corresponding author on reasonable request.

## Abbreviations

AA: Arachidonic acid
ACA: Anterior cerebral artery
BCA: Bicinchoninic acid
CCL2: C-C motif chemokine 2
DAB: 3,3′-Diaminobenzidine
DAPI: 4′,6-Diamidino-2-phenylindole dihydrochloride
DHA: Docosahexaenoic acid
DMEN: Dulbecco’s modified Eagle medium
EPA: Eicosapentaenoic acid
FFAR4: Free fatty acid receptor 4
GPCRs: G-protein coupled receptors
GPR120: G-protein coupled receptor 120
HMG-CoA: Hydroxymethylglutaryl-coenzyme A
IA: Intracranial aneurysm
IEL: Internal elastic lamina
IKKβ: Inhibitor of kappa B kinase subunit β
LPS: Lipopolysaccharide
MCP-1: Monocyte chemoattractant protein 1
NSAIDs: Non-steroid anti-inflammatory drugs
NF-κB: Nuclear factor-kappa B
OA: Olfactory artery
SAH: Subarachnoid hemorrhage
SDS-PAGE: Sodium dodecyl sulfate-poly-acrylamide gel electrophoresis
SMA: Smooth muscle α-actin
TAB1: Transforming growth factor-β activated kinase 1 binding protein
TAK1: Transforming growth factor-β activated kinase 1
TGF-β: Transforming growth factor-β
