# Supplementary figures and images for: Eicosapentaenoic acid prevents the progression of intracranial aneurysms in rats
Source: J Neuroinflammation. 2020 Apr 24;17:129. doi: 10.1186/s12974-020-01802-8 (PMC7181479; doi:10.1186/s12974-020-01802-8)

Fig.S1

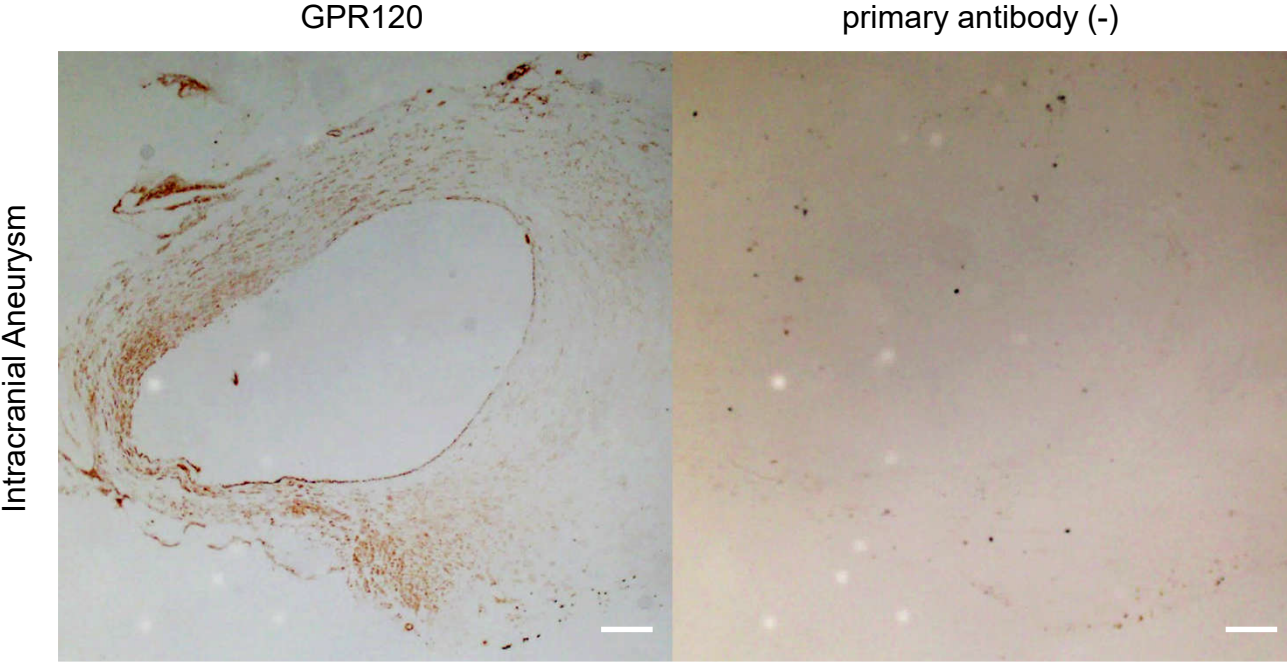

the same image as Figure 1

Fig.S2

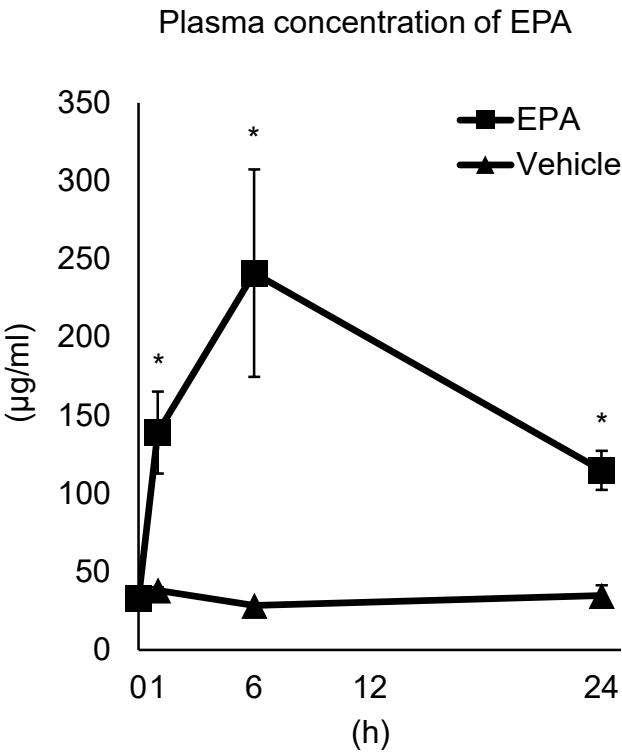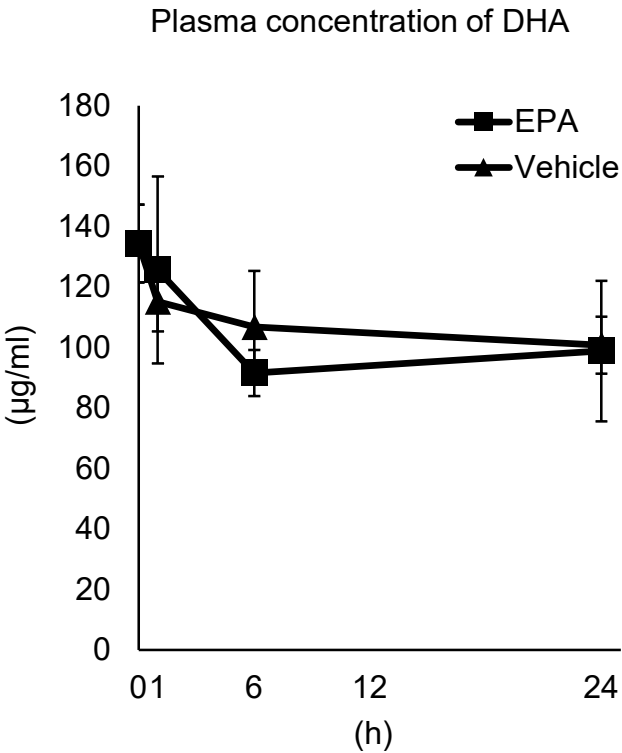

Fig.S3

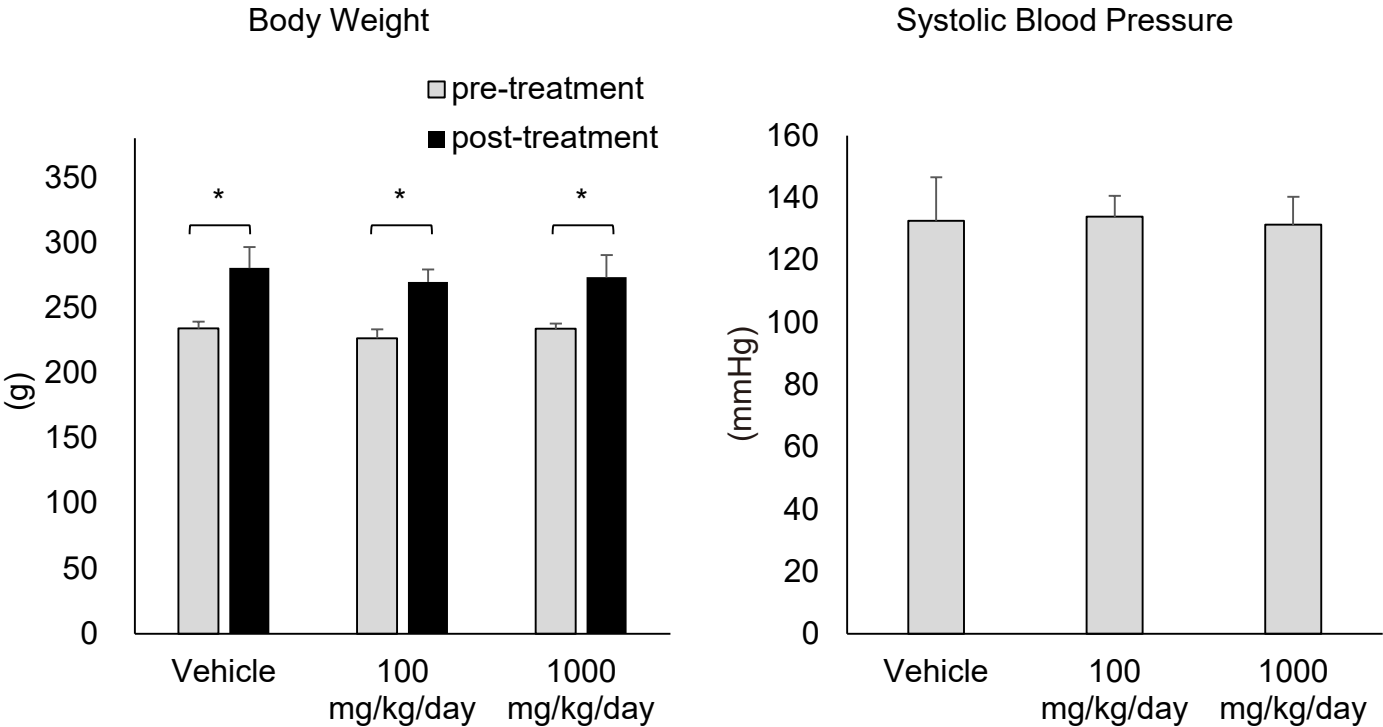

Fig.S4

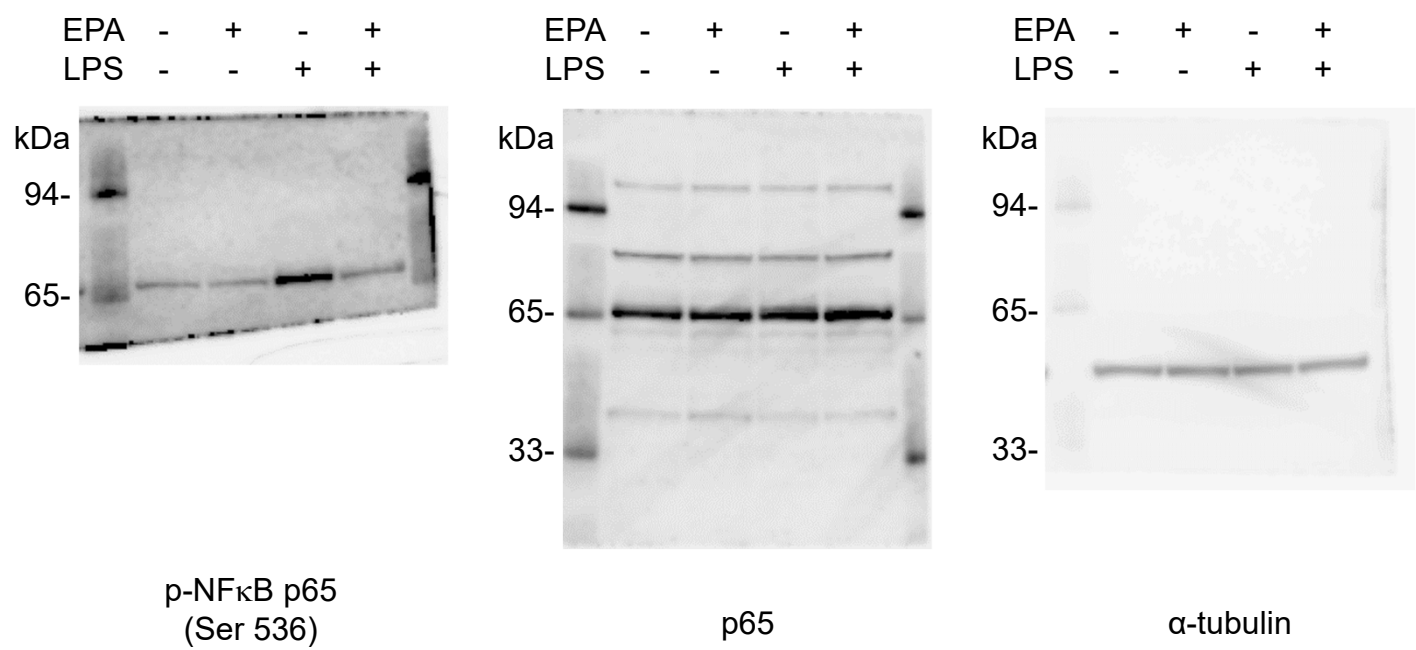

Fig.S5

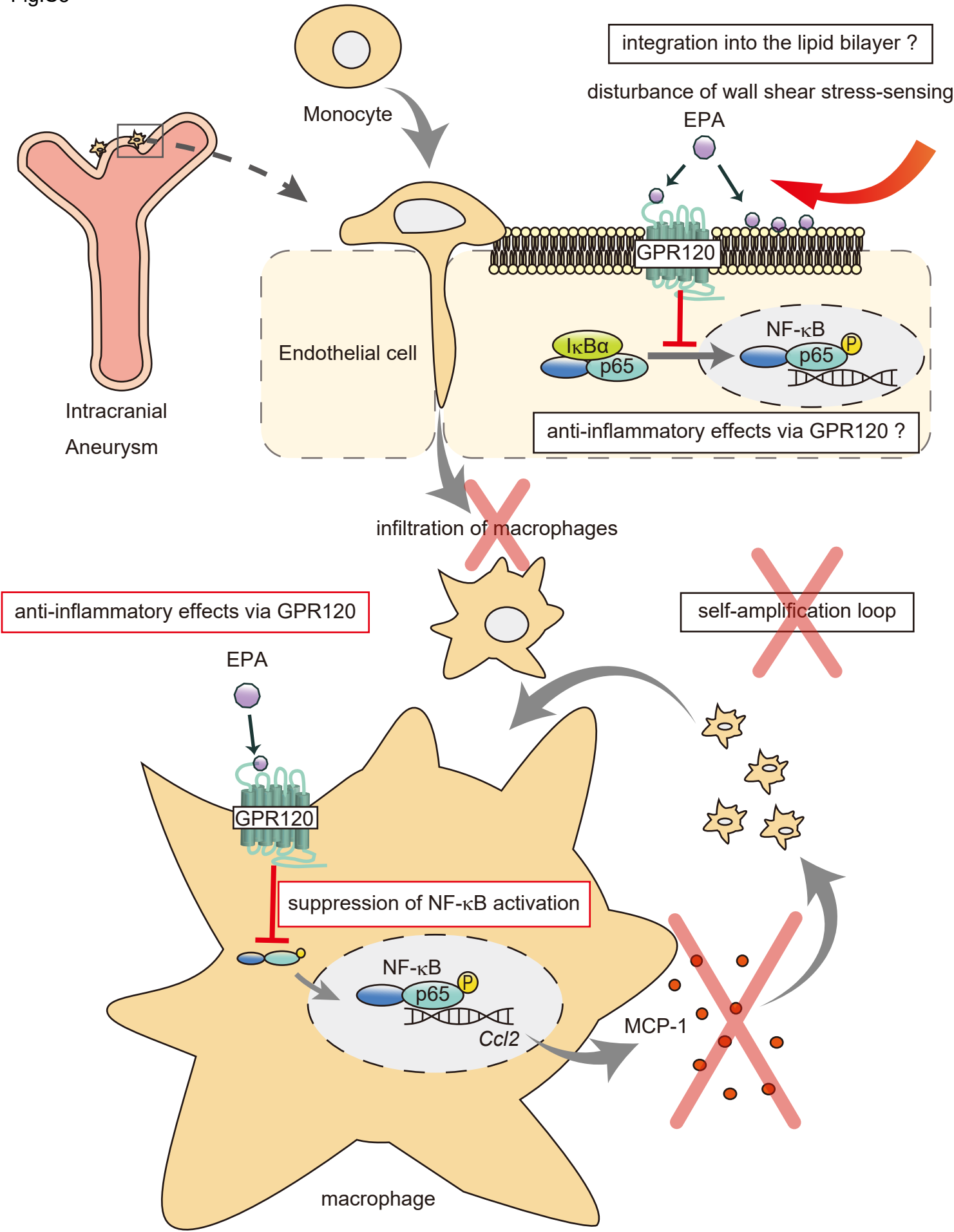

Supplement: Supplementary file 1 — Additional file 1: Fig. S1. GPR120 expression in human IA lesions. The representative image of immunostaining for GPR120 using human IA specimen is shown in the left panel. The image from immunostaining without a primary antibody for GPR120 is shown as a negative control study in the right panel. The image in the left panel is the same one used in Fig. 1. Bar; 200 μm. Fig. S2. Temporal change of the concentration of EPA and DHA in plasma of rats orally administered EPA. EPA (1000 mg/kg) was orally administered to rats and the concentration of EPA and DHA in plasma was measured at 1 h (n=3), 6 h (n=3) or 24 h (n=4) after the administration. Data represents mean ± SD. Statistical analysis was done by a Mann-Whitney test. *; p<0.05. Note that the plasma concentration of DHA was not increased by the administration of EPA. Fig. S3. Body weight and systemic blood pressure of rats treated with EPA. Rats were subjected to surgical manipulations to induce IAs and then given vehicle or EPA (100 or 1000 mg/kg/day) for 12 days. Body weight (the left panel) and systemic blood pressure (the right panel) were then measured (vehicle, n=9, 100 mg/kg/day, n=10, 1000 mg/kg/day, n=9). Data represents mean ± SD. Statistical analysis was done by a Kruskal-Wallis test. Fig. S4. Full-scanned images of western blot analysis in Fig. 6a. RAW264.7 cells were treated with EPA (300 μM) for 60 min and then stimulated with LPS (3.3 μg/ml) for additional 10 min. NF-κB activation was then assessed by western blot analysis using the whole cell lysate. The whole membranes of the western blot analysis presented in Fig. 6a are shown. Protein molecular weight markers are also displayed on both sides. Fig. S5. The graphical abstract of the suppressive effects of EPA on the progression of IAs. The one of the major mechanisms underlying the suppressive effect of EPA on the progression of IAs is the inhibition of inflammation by macrophages through interfering NF-κB activation via GPR120. Note the interrupti [file 12974_2020_1802_MOESM1_ESM.pdf]
